# Explainable artificial intelligence approaches for COVID-19 prognosis prediction using clinical markers

**DOI:** 10.1038/s41598-024-52428-2

**Published:** 2024-01-20

**Authors:** Krishnaraj Chadaga, Srikanth Prabhu, Niranjana Sampathila, Rajagopala Chadaga, Shashikiran Umakanth, Devadas Bhat, Shashi Kumar G S

**Affiliations:** 1 Department of Computer Science and Engineering, Manipal Institute of Technology, Manipal Academy of Higher Education, Manipal, Karnataka India; 2 Department of Biomedical Engineering, Manipal Institute of Technology, Manipal Academy of Higher Education, Manipal, Karnataka India; 3 Department of Mechanical and Industrial Engineering, Manipal Institute of Technology, Manipal Academy of Higher Education, Manipal, Karnataka India; 4 Department of Medicine, Dr. TMA Hospital, Manipal Academy of Higher Education, Manipal, Karnataka India; 5 Department of Electronics and Communication Engineering, Manipal Institute of Technology, Manipal Academy of Higher Education, Manipal, Karnataka India

**Keywords:** Predictive markers, Prognostic markers, Biomedical engineering

## Abstract

The COVID-19 influenza emerged and proved to be fatal, causing millions of deaths worldwide. Vaccines were eventually discovered, effectively preventing the severe symptoms caused by the disease. However, some of the population (elderly and patients with comorbidities) are still vulnerable to severe symptoms such as breathlessness and chest pain. Identifying these patients in advance is imperative to prevent a bad prognosis. Hence, machine learning and deep learning algorithms have been used for early COVID-19 severity prediction using clinical and laboratory markers. The COVID-19 data was collected from two Manipal hospitals after obtaining ethical clearance. Multiple nature-inspired feature selection algorithms are used to choose the most crucial markers. A maximum testing accuracy of 95% was achieved by the classifiers. The predictions obtained by the classifiers have been demystified using five explainable artificial intelligence techniques (XAI). According to XAI, the most important markers are c-reactive protein, basophils, lymphocytes, albumin, D-Dimer and neutrophils. The models could be deployed in various healthcare facilities to predict COVID-19 severity in advance so that appropriate treatments could be provided to mitigate a severe prognosis. The computer aided diagnostic method can also aid the healthcare professionals and ease the burden on already suffering healthcare infrastructure.

## Introduction

The COVID-19 began in late 2019 and caused a significant uproar worldwide^1^. Most patients experienced mild-moderate symptoms such as cough, cold, myalgia, sore throat, muscle pain, nausea, loss of taste/smell and headaches. However, people also developed severe symptoms such as accurate respiratory disorder syndrome (ARDS), severe hypoxia and multi-organ failure and succumbed to this deadly disease^2^. As of today, the virus is still spreading, and new mutations are being created. Cytokine storm manifests in COVID-19 patients, distinguished by an enormous release of cytokines such as IL-6 and IL-1. This condition has led to the immune system attacking itself and has caused deaths in many Sars-Cov-2 patients^3^.

The severe symptoms of COVID-19 have decreased after the introduction of vaccines^4^. However, some COVID-19 patients are still vulnerable to severe prognoses^3^. Older patients and people with comorbidities such as hypertension, diabetes, cancer etc., are still at risk. It is crucial to identify these patients early so that appropriate medications and treatments can be provided to them to avoid unnecessary casualties. A few drugs have been created and shown to prevent the onset of severe COVID-19 symptoms^3^. These medicines must be administered during the illness's initial stages to be effective.

Artificial Intelligence (AI) applications have been extensively utilized in the healthcare sector^5–7^. Diagnostic and prognostic models, decision support systems and predictive modelling are being developed to assist healthcare professionals using machine learning (ML). The above technologies are also being used in the fight against COVID-19^8–10^. Explainable artificial intelligence (XAI) makes the models more transparent and understandable. The reasoning behind a patient prediction can be visually represented using XAI. It has also been utilized in various domains such as finance, engineering, pharmacy, medicine and commerce.

A few markers such as c-reactive protein (CRP), D-Dimer, lactate dehydrogenase (LDH), neutrophil to lymphocyte ratio (NLR) and Ferritin are known to change excessively before the actual onset of the severe symptoms^2^. Machine learning models can be deployed using the markers to predict COVID-19 severity in advance. The early detection of patients with poor prognoses and the development of reliable forecasting techniques that are simple to use in routine clinical practice are crucial for ensuring the highest level of treatment in clinics.

Several researchers have utilized machine learning to predict COVID-19 severity using hematological parameters. Huyut^11^ developed an automatic decision support system to predict mild and severe coronavirus patients. The dataset consisted of 194 severe and 4010 mild patients. Twenty-nine markers were considered, and the local weighted algorithm obtained a maximum accuracy of 97.86%. Wendland et al.^12^ used classifiers to predict severe COVID-19 cases. They were able to predict the severity status with an AUC of 0.918. The most important markers in this study were CRP and blood sugar levels. A COVID-19 severity prediction model was developed by Nguyen et al.^13^. Two hundred sixty-one patients from Vietnam were considered for this research. The random forest obtained a accuracy of 97%. The best prognostic markers were CRP, IL-6, dyspnea, D-Dimer and ferritin. A nature-inspired was developed to predict COVID-19 severity^14^. The study used details of 65,000 patients, which consisted of twenty-six features. A variant of the artificial bee colony algorithm was used for feature selection. Among all the algorithms, the support vector machine obtained an accuracy of 96%. The model categorized the patients into mild, moderate and severe. Laatifi et al.^15^ used two explainable AI techniques to predict COVID-19 severity. Eighty-seven patients were considered in this study. Shapley additive values (SHAP) and local interpretable model-agnostic explanations (LIME) were used to make the models understandable. The most critical cytokine markers are VEGF-A and IL-7.

COVID-19 vaccines have been successful in preventing severe COVID-19 in most patients. However, a small part of the population still experiences severe symptoms. It is of utmost importance to prevent the onset of severe prognosis in these patients. The machine learning models can be beneficial in predicting the same.

The above studies show that COVID-19 severity could be predicted effectively using clinical and laboratory markers. The main objective of this research is to forecast the severity of a COVID-19 patient. The other contributions are given below:

- Descriptive statistical analysis of the data has been conducted to understand various trends and patterns in the data.
- Fourteen feature selection methods including nature-inspired algorithms have been used to choose the most important markers.
- Machine learning models including bagging, boosting, voting and stacking have been used to predict COVID-19 severity. The classifiers have been further compared to with the state-of-the-art deep learning models such as deep neural network (DNN), one-dimensional convolutional neural network (1D-CNN) and Long short-term memory (LSTM).
- Five XAI techniques have been used to interpret the predictions such as SHAP, LIME, Eli5, QLattice and Anchor.
- Further discussion about crucial COVID-19 prognostic markers from a medical perspective.

The reminder of the paper is structured as follows. Materials and methods are described in “Methods” section. Extensive explanation of the results is made in “Results” section. The discussion of the results obtained is made in “Discussion” section. The article concludes in “Conclusion” section.

## Methods

### Description of the dataset

The COVID-19 datasets were obtained from two Hospitals in India: Dr TMA Pai Hospital and Kasturba Medical College. The Manipal Academy of Higher Education has provided ethical clearance to conduct this research (IEC:613/2021). The patients have been completely anonymized in this study. COVID-19 patients who were tested between September 2021 and December 2021 have been considered in this study. Only patients above eighteen years of age have been included. Records of 899 patients have been utilized to train the machine learning models. The dataset included 599 non-severe patients and 300 severe patients. All patients whose condition deteriorated and required admission to the intensive care unit (ICU) and if the respiratory rate > 30/minute or SpO2 < 90% (World Health Organization standards) were grouped as severe cases^16^. Thirty-two clinical parameters were considered in this study (31—continuous and one categorical). The clinical markers chosen are tabulated in Table 1.

**Table 1:** Attributes chosen in this study.

Sl. No.	Clinical marker	Marker description	Unit	Sl. No.	Clinical marker	Marker description	Unit
1	Patient file number	File number of a COVID-19 patient		18	Creatinine	It consists of muscle creatine	mg/dL
2	Age	Age of a patient in years		19	Sodium	Sodium content present in the body	mmol/L
3	Gender	Sex of a patient		20	Potassium	Potassium content present in the body	mmol/L
4	SpO2	Oxygen saturation in the blood	%	21	Total Bilirubin (T. Bilirubin)	Direct and indirect bilirubin combine to formal total bilirubin	mg/dL
5	Pulse	The number of heart beats per minute		22	Direct Bilirubin (D. Bilirubin)	Bilirubin content which could be removed from the body	mg/dL
6	Respiratory rate	Number of breaths taken per minute		23	Aspartate aminotransferase (AST)	An enzyme produced by the liver	IU/L
7	Hemoglobin	It delivers oxygen to all the organs and tissues	gram/dL	24	Alanine transaminase (ALT)	An enzyme produced by the liver	IU/L
8	Hematocrit	It is the percentage of red blood cells in the blood	%	25	Alkaline phosphatase (ALP)	An enzyme produced by the liver	IU/L
9	Total white blood cells (TWBC)	They are part of the immune system. They help in fighting infections	10^3^/microliter	26	Total protein	It shows how much protein the liver is able to make	g/dL
10	Neutrophil	They belong to the white blood cells category	%	27	Albumin	A protein made by the liver	g/dL
11	Lymphocyte	They belong to the white blood cells category	%	28	HbA1c	Average blood sugar level in the last two to three months	%
12	Neutrophil to lymphocyte ration (NLR)	Number of neutrophils to the number of lymphocytes	%	29	C reactive protein (CRP)	A protein produced by the liver. Elevated levels of CRP indicate an infection	mg/L
13	Monocyte	They belong to the white blood cells category	%	30	D-Dimer	A protein which is formed when the blood clot dissolves	μg/mL
14	Eosinophil	They belong to the white blood cells category	%	31	Ferritin	It indicates iron content in the body	μg/L
15	Platelet	Platelets help in forming blood clots	thousand/μL	32	Lactate dehydrogenase (LDH)	It is an enzyme which helps is storing energy	U/L
16	Basophil	They belong to the white blood cells category	%	33	Label	Non-Severe/Severe	
17	Urea	It consists of dietary protein and tissue protein	mg/dL				

### Data pre-processing

Pre-processing of the dataset is critical in machine learning. Missing values are imputed, categorical attributes are encoded, continuous values are scaled, data balancing is performed, and unnecessary attributes are dropped. In order to make sure that there are as few missing values as possible, we chose patients who completed the most clinical tests when gathering data. A few missing values in the dataset were replaced by their respective median. The “gender” attribute (categorical) had no missing values. Descriptive statistical analysis was conducted using the open-source statistical software Jamovi. Some statistical parameters utilized are described in Table 2.

**Table 2:** Descriptive statistical parameters for the COVID-19 dataset.

	Label	Number of instances	Marker mean	Marker median	Standard deviation	Inter quartile range	Minimum value	Maximum value	25th percentile	50th percentile	75th percentile
Age	Non-severe	599	47.35	48	19.3877	30	18	99	32	48	62
	Severe	300	59.102	61	15.5633	21	18	92	49	61	70
SpO2	Non-severe	599	96.828	97	1.8738	2	88	100	96	97	98
	Severe	300	90.764	94	10.0901	7	35	100	90	94	97
PR(Pulse)	Non-severe	599	85.964	85	14.4364	18	22	150	76	85	94
	Severe	300	91.61	90	17.614	20	18	185	80	90	100
Respiratory rate	Non-severe	599	20.783	20	1.6421	2	12	30	20	20	22
	Severe	300	23.441	22	5.0726	3	13	52	21	22	24
Hb (Hemoglobin)	Non-severe	599	13.179	13.1	1.8338	2.3	3.7	17.4	12.2	13.1	14.5
	Severe	300	12.493	12.7	2.248	2.8	4.5	18.7	11.2	12.7	14
PCV% (Hematocrit)	Non-severe	599	38.801	39	5.5506	6.9	9	50	36	39	42.9
	Severe	300	37.306	37.8	6.9346	8.075	11.9	92	33.625	37.8	41.7
TWBC	Non-severe	599	5.587	5.3	2.0146	2.2	0.8	14.1	4.3	5.3	6.5
	Severe	300	9.858	8.1	6.485	6.7	0.2	46.8	5.6	8.1	12.3
Neutrophil	Non-severe	599	61.563	62	11.5501	15	25	95	55	62	70
	Severe	300	78.954	80	11.5686	16.425	10.64	98	71.575	80	88
Lymphocyte	Non-severe	599	27.331	26	11.0344	13	2.1	91	20	26	33
	Severe	300	12.578	10.65	8.6193	10.875	1.1	60	6.125	10.65	17
NLR	Non-severe	599	2.599	2	2.9444	2	1	34	1	2	3
	Severe	300	11.102	7	12.2208	9	1	87	4	7	13
Monocyte	Non-severe	599	9.605	9	3.6886	5	0.8	21	7	9	12
	Severe	300	6.795	6.45	3.4439	4.9	0.2	18.1	4.1	6.45	9
Eosinophil	Non-severe	599	1.157	0.5	1.8009	1.3	0	13.9	0.1	0.5	1.4
	Severe	300	0.457	0.2	0.9674	0.2	0	9	0.1	0.2	0.3
Platelet	Non-severe	599	236.392	223	86.1237	98	67	602	180	223	278
	Severe	300	249.096	225	114.9165	115.75	50	920	180.25	225	296
Basophil	Non-severe	599	0.433	0.4	0.3403	0.2	0	4	0.3	0.4	0.5
	Severe	300	0.256	0.2	0.2336	0.1	0	2.6	0.2	0.2	0.3
Urea	Non-severe	599	23.152	21	13.1015	10	6	118	16	21	26
	Severe	300	44.262	32	41.2207	28	3	243	21	32	49
Creatinine	Non-severe	599	0.874	0.8	0.3435	0.3	0.3	4.7	0.7	0.8	1
	Severe	300	1.414	0.905	1.7923	0.5975	0.2	17.7	0.702	0.905	1.3
Sodium	Non-severe	599	137.262	138	4.5147	5	111	148	135	138	140
	Severe	300	134.617	135	5.7938	7	111	167	131	135	138
Potassium	Non-severe	599	4.122	4.1	0.4673	0.6	2.9	5.7	3.8	4.1	4.4
	Severe	300	4.31	4.2	0.7323	0.875	2.1	8	3.8	4.2	4.675
T. Bilirubin	Non-severe	599	0.468	0.4	0.2421	0.3	0.1	1.6	0.3	0.4	0.6
	Severe	300	0.755	0.595	0.9477	0.4	0.146	11	0.4	0.595	0.8
D.Bilirubin	Non-severe	599	0.191	0.2	0.0985	0.1	0.07	0.7	0.1	0.2	0.2
	Severe	300	0.4	0.24	0.7963	0.2	0.04	12	0.17	0.24	0.37
AST	Non-severe	599	35.282	29	22.9893	20	5	229	22	29	42
	Severe	300	62.552	47.5	52.5474	42.75	4.8	411	31	47.5	73.75
ALT	Non-severe	599	32.291	25	23.9823	22	5	180	17	25	39
	Severe	300	50.791	37	56.2449	33.75	3.5	659	22.25	37	56
ALP	Non-severe	599	78.867	77	26.7934	28	17	200	62	77	90
	Severe	300	100.853	85.5	57.4287	45.5	5	418	66.25	85.5	111.75
Protein	Non-severe	599	7.163	7.2	0.4714	0.1	4.2	8.5	7.1	7.2	7.2
	Severe	300	6.752	6.9	0.7361	0.5	3.2	12.4	6.5	6.9	7
Albumin	Non-severe	599	4.267	4.3	0.3869	0.1	3	7	4.2	4.3	4.3
	Severe	300	3.631	3.7	0.5154	0.5	1.02	5.3	3.4	3.7	3.9
HbA1c	Non-severe	599	6.12	5.6	1.5407	1	4	14	5.2	5.6	6.2
	Severe	300	7.165	6.6	1.9296	2.1	4.3	18.2	5.9	6.6	8
CRP	Non-severe	599	16.847	7	27.8004	17	0.3	223	2	7	19
	Severe	300	96.345	78	78.7297	97	0.47	350	37	78	134
D-Dimer	Non-severe	599	0.383	0.2	0.6899	0.19	0.1	9.12	0.16	0.2	0.35
	Severe	300	1.82	0.8	2.271	1.5475	0.1	14	0.453	0.8	2
Ferritin	Non-severe	599	301.776	197	330.1534	273	1.6	2000	89	197	362
	Severe	300	830.517	718	594.9111	774.25	10.43	2000	347	718	1121.25

**Figure 1:**
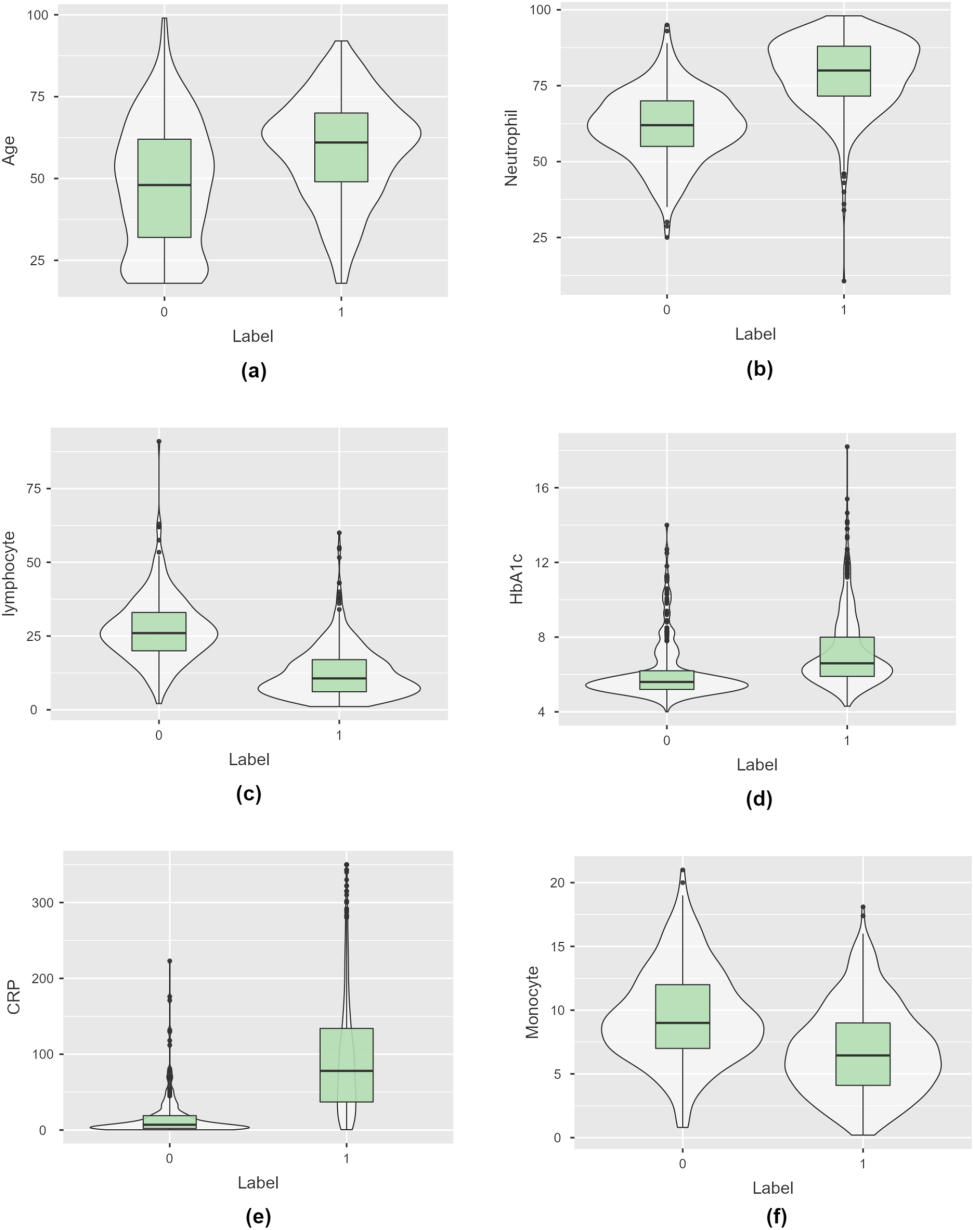
Violin plots for some of the markers. (**a**) Age (**b**) Neutrophil (**c**) Lymphocyte (**d**) HbA1c (**e**) CRP (**f**) Monocyte.

Violin plots were used to find interesting patterns in the dataset, as shown in Fig. 1. From the figure, it can be seen that the median age was elevated in the severe COVID-19 cohort. Further, markers such as Neutrophils, HbA1c and CRP were elevated in severe patients. The lymphocytes and monocytes count decreased in the severe COVID-19 cohort.

**Figure 2:**
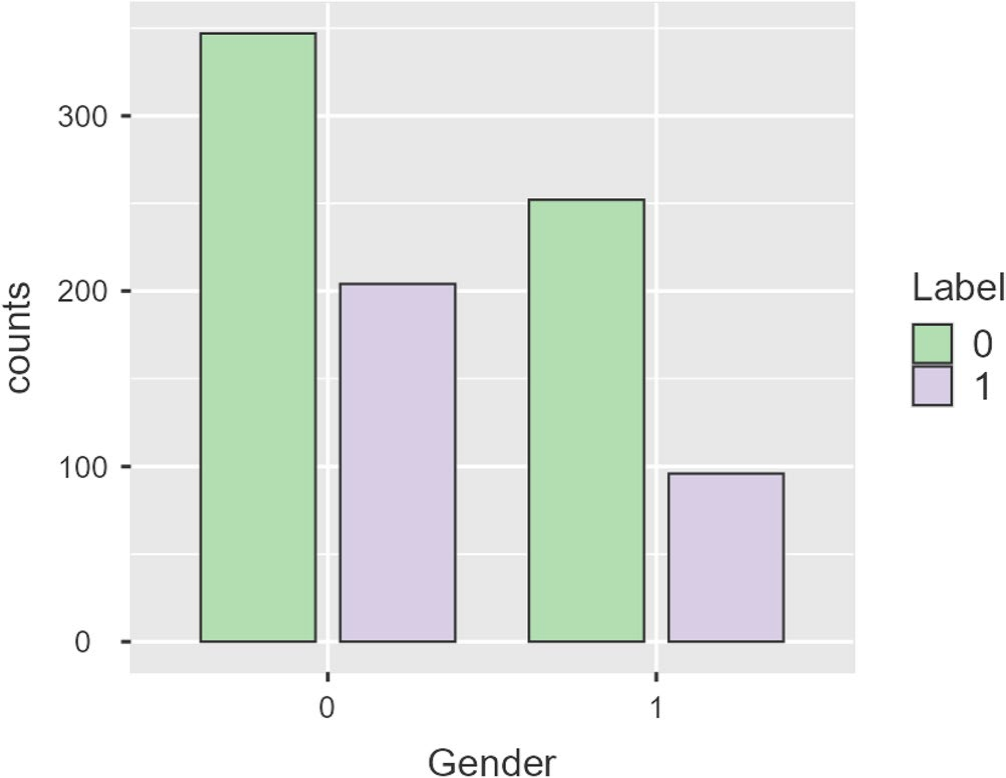
Frequency distribution of the gender attribute.

The frequency of the “gender” attribute for severe/non-severe COVID-19 patients is described using a bar plot in Fig. 2. There were 347 male and 252 female patients in the non-severe cohort. There were 204 male patients and 96 female patients in the severe cohort.

In machine learning analysis, categorical values must be encoded since the classifiers do not handle text values. Several encoding techniques exist in machine learning^17^. In this study, we used the one-hot encoding technique to encode the “Gender” attribute^18^. This encoding mechanism solves the problem of ordinality, which can happen in categorical variables. Data scaling was performed using the standardization method^19^. When there is a considerable discrepancy in data points, the accuracy decreases. The classifiers also favour parameters with higher values, regardless of the units considered. Normalization and standardization are the two approaches utilized to scale the datasets in machine learning. Standardization was chosen in this study since they are better with outliers. The dataset was then split into training and testing in the ratio (80:20). There was a significant imbalance in the dataset. The number of severe COVID-19 cases was almost half compared to non-COVID-19 cases. The results obtained for the unbalanced data are completely biased since the models favour the majority classes. Hence, we used the oversampling technique called Borderline Synthetic Minority Oversampling Technique (SMOTE) to balance the training dataset^20^. This algorithm generates new synthetic samples using the K-nearest algorithm. The borderline cases are also handled well using the above technique. Under-sampling was not preferred in this study since we did not want to lose interesting trends and patterns. Further, the testing data was not balanced to protect data integrity.

**Figure 3:**
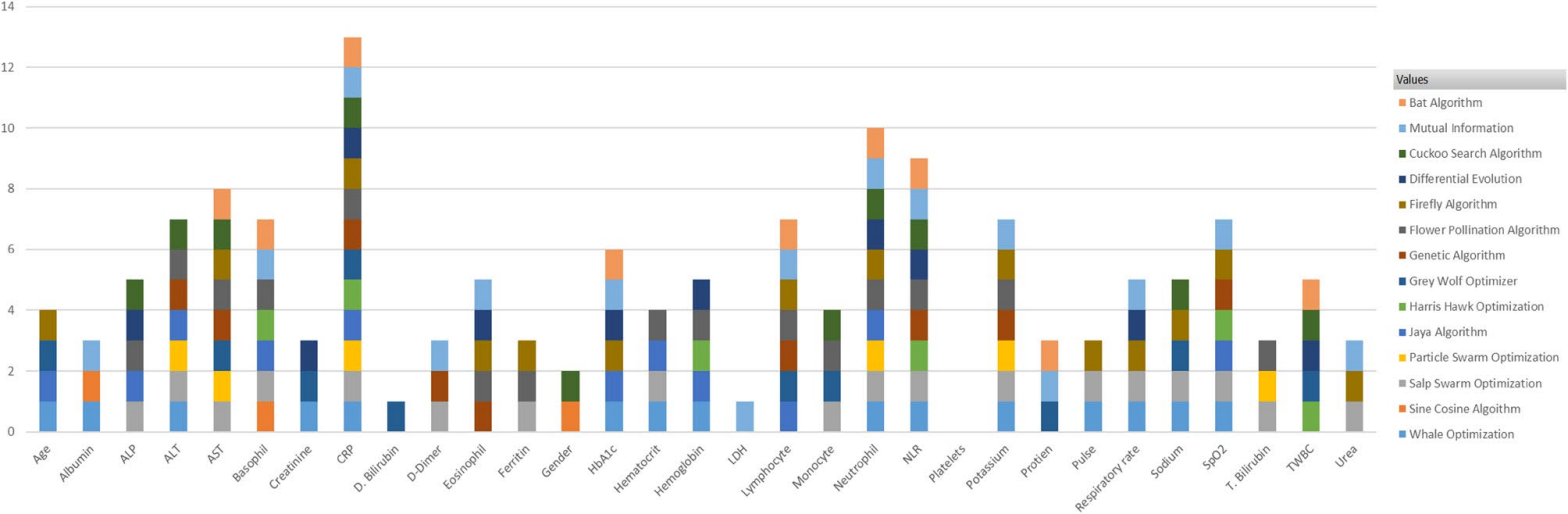
Markers chosen by the feature selection methods.

Fourteen feature selection methods were used to choose the most important markers. Several metaheuristic nature-inspired algorithms have been utilized in this study. Feature selection is essential in machine learning since the classifiers perform better when removing redundant features. In this article, we have chosen multiple nature inspired algorithms. They have several advantages over traditional feature selection techniques. They are known for their global optimization, robustness, scalability, parallelism, adaptability, simplicity and stochasticity. Table 3 describes the features chosen by each algorithm. Among all the algorithms, the salp swarm optimization chose the maximum number of features (18). The whale optimization algorithm, flower pollination algorithm and mutual information chose 15 features. The sine cosine algorithm chose the minimum number of features (3). The Harris Hawk’s optimization and particle swarm optimization chose six features each. The markers chosen by the feature selection techniques are also described in Fig. 3. CRP was the most chosen feature since thirteen algorithms have included it. This was followed by neutrophils, NLR and AST, which were chosen 10, 9 and 8 times, respectively. The marker platelets were not chosen by any algorithm.

**Table 3:** Feature selection using several algorithms.

Sl.no	Feature selection method used	Number of features chosen	Features chosen
1	Whale Optimization (WO)^21^	15	Age, SpO2, Pulse, Respiratory rate, Hemoglobin, Haemtocrit, Neutrophil, NLR, Creatinine, Sodium, Potassium, ALT, Albumin, HbA1c and CRP
2	Sine Cosine Algorithm (SCA)^22^	3	Gender, Basophil and Albumin
3	Salp Swarm Optimization (SSO)^23^	18	SpO2, Pulse, Respiratory rate, Hematocrit, Neutrophil, NLR, Monocyte, Basophil, Urea, Sodium, Potassium, T. Bilirubin, AST, ALT, ALP, CRP, D-Dimer and Ferritin
4	Particle Swarm Optimization (PSO)^24^	6	Neutrophil, Potassium, T. Bilirubin, AST, ALT and CRP
5	Jaya Algorithm (JA)^25^	11	Age, SpO2, Hemoglobin, Hematocrit, Neutrophil, Lymphocyte, Basophil, ALT, ALP, HbA1c and CRP
6	Harris Hawks Optimization (HHO)^26^	6	SpO2, Hemoglobin, TWBC, NLR, Basophil and CRP
7	Grey Wolf Optimizer (GWO)^27^	10	Age, TWBC, Lymphocyte, Monocyte, Creatinine, Sodium, D. Bilirubin, AST, Protein and CRP
8	Genetic Algorithm (GA)^28^	9	SpO2, Lymphocyte, NLR, Eosinophil, Potassium, AST, ALT, CRP and D-Dimer
9	Flower Pollination Algorithm (FPO)^29^	15	Hemoglobin, Hematocrit, Neutrophil, Lymphocyte, NLR, Monocyte, Eosinophil, Basophil, Potassium, T. Bilirubin, AST, ALT, ALP, CRP and Ferritin
10	Firefly Algorithm (FA)^30^	14	Age, SpO2, Pulse, Respiratory rate, Neutrophil, Lymphocyte, Eosinophil, Urea, Sodium, Potassium, AST, HbA1c, CRP and Ferritin
11	Differential Evolution (DE)^31^	10	Respiratory rate, Hemoglobin, TWBC, Neutrophil, NLR, Eosinophil, Creatinine, ALP, HbA1c and CRP
12	Cuckoo Search Algorithm (CSA)^32^	10	Gender, TWBC, Neutrophil, NLR, Monocyte, Sodium, AST, ALT, ALP and CRP
13	Bat Algorithm (BA)^33^	9	TWBC, Neutrophil, Lymphocyte, NLR, Basophil, AST, Protein, HbA1c and CRP
14	Mutual Information (MI)^34^	15	Albumin, NLR, Protein, CRP, D-Dimer, SpO2, Lymphocyte, LDH, Basophil, Eosinophil, Urea, Neutrophil, Respiratory rate and HbA1c

### Machine learning concepts

**Figure 4:**
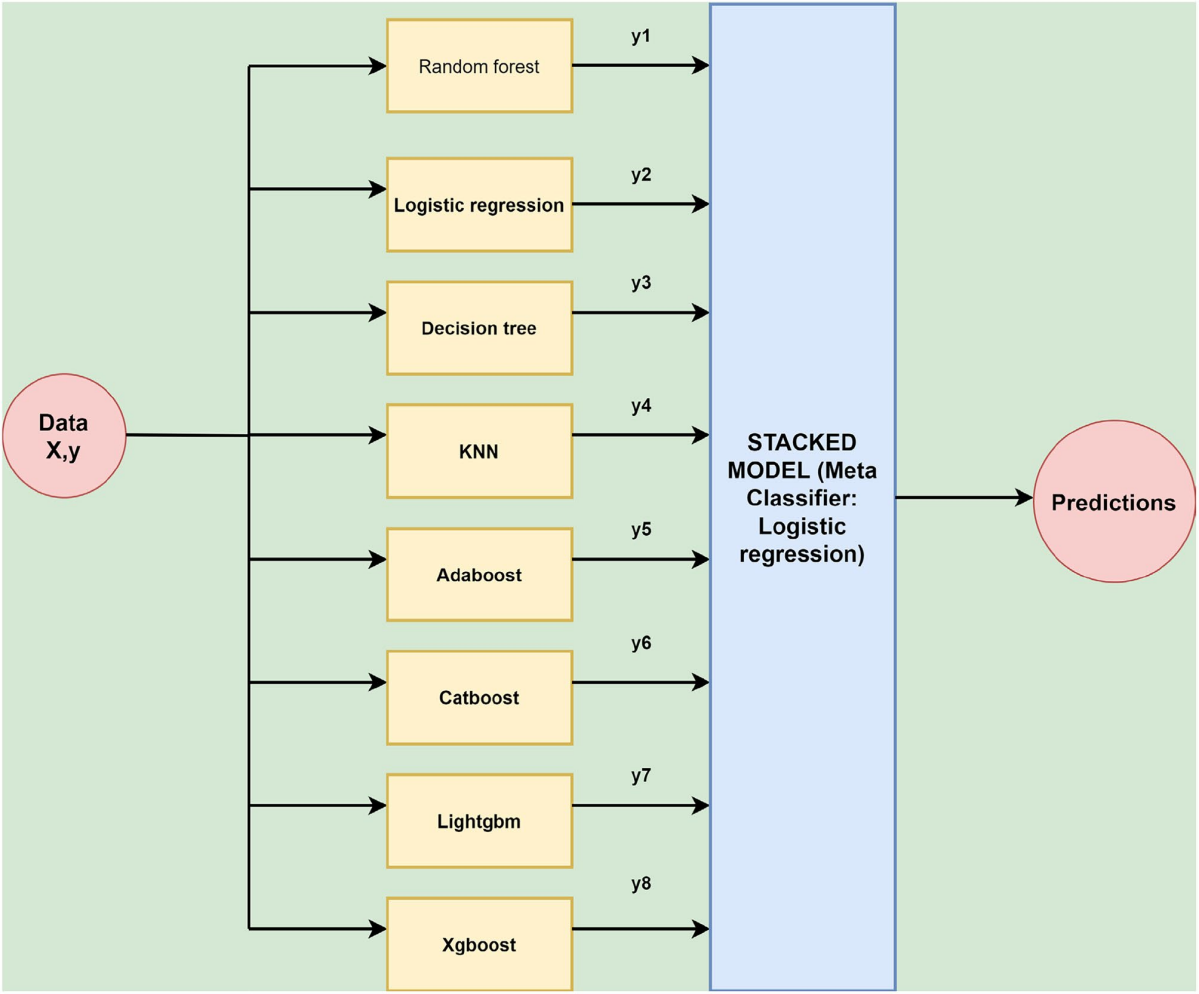
Stacking methodology used in this research.

Machine learning is a form of artificial intelligence that enables software programs to forecast predictive outcomes using past information as input. Several ML classifiers have been used in this study, such as random forest, decision tree, logistic regression, K nearest neighbors, catboost, adaboost, xgboost, lightgbm, stacking and voting algorithms. Stacking combines the result of multiple baseline models^35^. The stacking architecture consists of a classifier incorporating the initial model’s predictions. Aggregation of the models are performed based on their weights, improving the model’s accuracy. The meta-learner becomes a crucial factor in stacking. Logistic regression was the meta-learner used in this research. The stacking architecture is described in Fig. 4.

**Figure 5:**
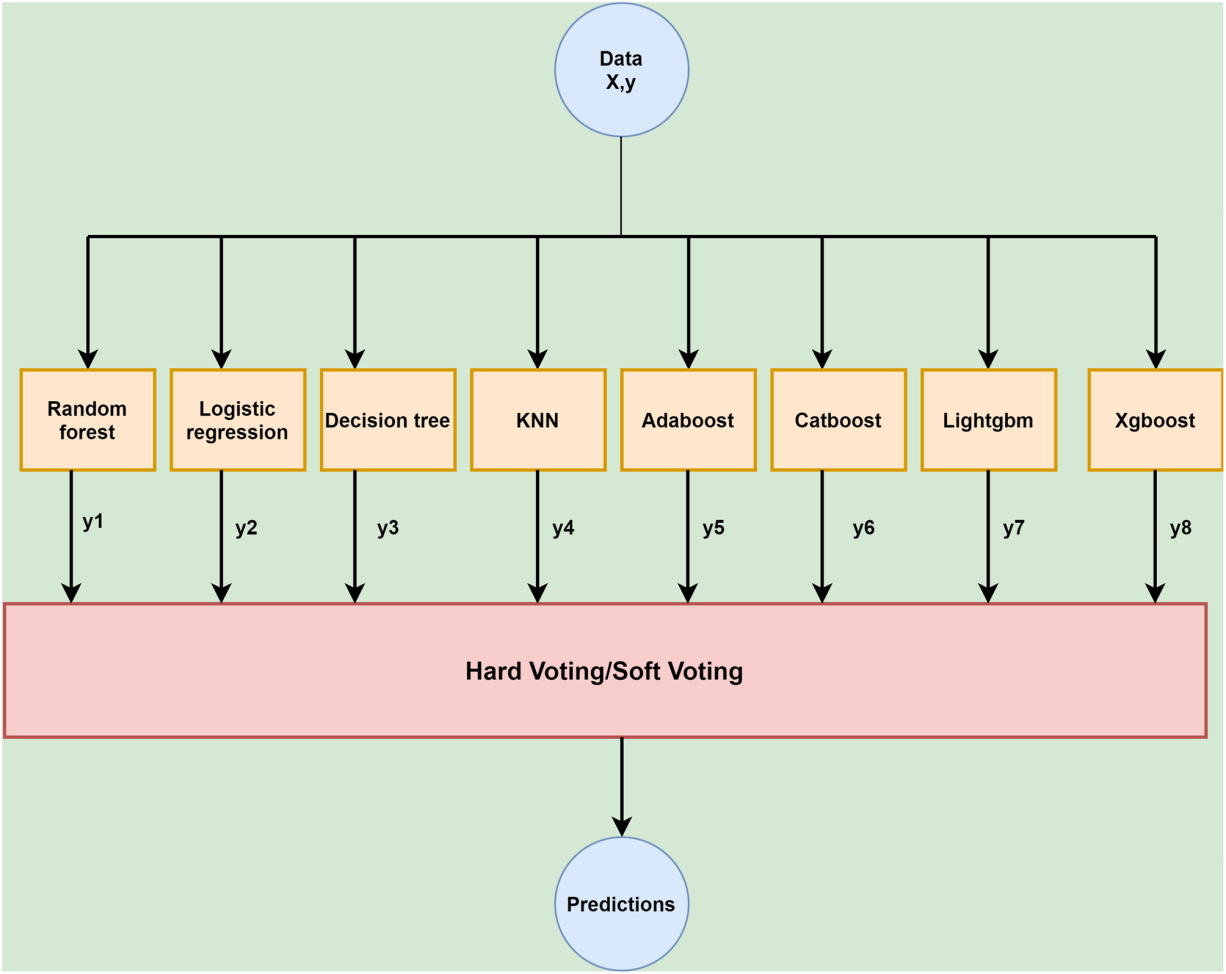
Voting methodology used in this research.

A voting classifier gathers training data from a large ensemble of classifiers, and predictions are made according to the class with the highest probability. It uses the concept of majority voting^36^. The voting algorithm is of two types: Hard-voting and soft-voting. The maximum number of votes is considered in hard-voting irrespective of the weights^37^. “Average probability” predicts the outcome of soft-voting^38^. The voting architecture is described in Fig. 5.

Further, the data was subjected to a fivefold cross validation technique. Here, various subsets of data are trained to validate the model efficiency. The input data is divided into five equal groups. Four groups are used for training, while the fifth group is used for testing using various permutations and combinations in cross-validation. Hyperparameter tuning was performed to choose the best parameters using the grid search method. The performance of a classifier depends upon the hyperparameters chosen. Grid search automates the hyperparameter tuning and provides the best values as output.

We have chosen several classification and loss metrics to evaluate the models in this study. These include precision, recall, accuracy, F1-score, area under curve (AUC), average precision (AP), Mathew’s correlation, log loss, Jaccard score and hamming loss. Emphasis has been given to precision and recall since they focus on false-positive and false-negative cases.

In this research, three state-of-the-art deep learning models have been tested. They are DNN, 1D-CNN and LSTM. A DNN consists of multiple input, hidden and output layers^39^. The essential function of a deep neural network is to take input, process them through more sophisticated computations, and predict results. CNNs are primarily used for image classification. However, 1D-CNN models have also been highly influential in classifying tabular data^40^. LSTMs are highly used in sequence prediction problems^41^. Three types of gates are considered in LSTM: input gate, output gate, and forget gate. LSTMs have proven to be highly efficient in handling time series data.

**Figure 6:**
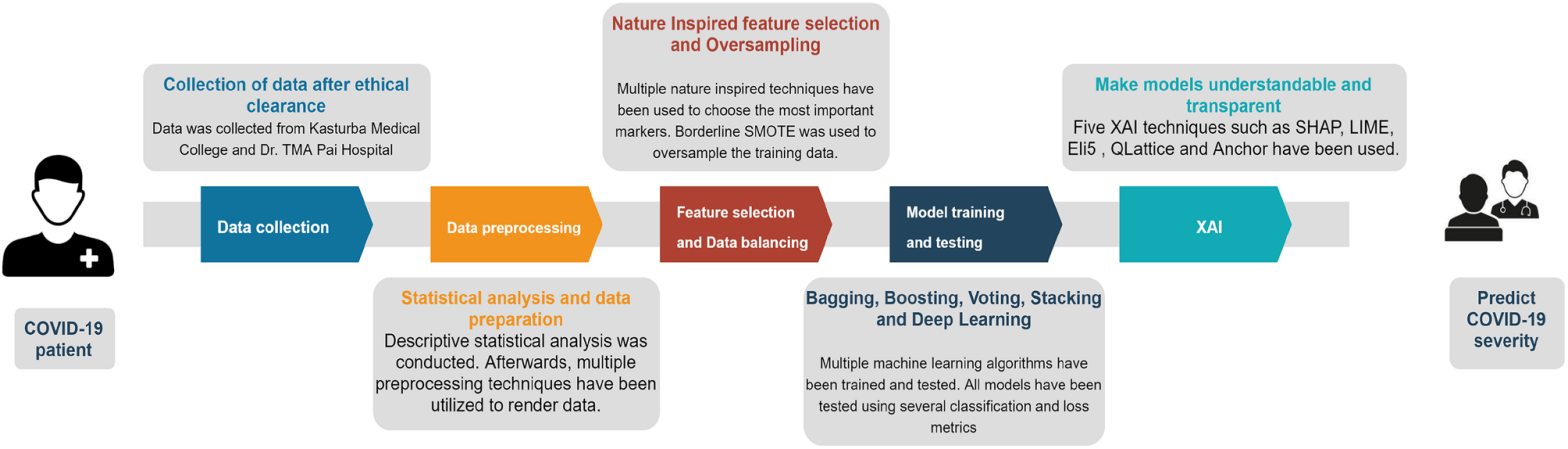
Machine learning methodology used in this research.

After training and testing the ML and DL models, five XAI techniques have been used to demystify the predictions. The results obtianed by the XAI techniques are in the form of graphs and tables, which can be easily understood by the ML users. The entire process-flow of this study is described in Fig. 6.

### Ethical approval

Ethical clearance has been obtained to collect patient data from Manipal Academy of Higher Education ethics committee with id IEC: 613/2021. The need for informed consent was waived by the ethics committee/Institutional Review Board of Manipal Academy of Higher Education, because of the retrospective nature of the study. All methods were carried out in accordance with relevant guidelines and regulations.

## Results

### Model testing

In this research, multiple machine learning and deep learning classifiers have been trained and tested to predict COVID-19 severity. The precision obtained by the models for various feature selection techniques is tabulated in Table 4. We emphasized the stacking and voting classifiers since they combine multiple models. From the table, it can be seen that the stacked model obtained the maximum precision of 94% after using mutual information. The soft-voting and hard-voting obtained a precision of 94% each. The bat algorithm performed well too. The stack, hard-voting and soft-voting classifier obtained a precision of 91%, 91% and 90%, respectively. The flower pollination algorithm was also efficient. The stack, hard-voting and soft-voting obtained a precision of 87%, 86% and 84%, respectively. The precision obtained for the stack, hard-voting and soft-voting after using the Jaya algorithm was 87%, 90% and 89%, respectively.

**Table 4:** Precision obtained by the classifiers for various feature selection methods (In %).

Algorithm	WO	SCA	PSO	JA	HHO	GWO	GA	FPO	FA	DE	CSA	BA	MI
Random forest	84	84	81	87	91	82	86	89	82	83	82	92	91
Logistic regression	81	75	78	81	82	79	81	84	83	81	80	83	83
Decision Tree	77	81	73	87	79	78	84	78	77	75	72	86	84
KNN	75	81	75	77	77	82	78	78	78	78	76	78	77
Adaboost	81	86	75	87	90	82	85	87	81	83	82	89	93
Catboost	86	86	76	88	92	82	86	88	82	86	82	90	94
Lightgbm	86	83	79	88	90	84	83	88	82	84	82	90	93
Xgboost	84	85	82	89	92	84	86	86	82	85	85	91	95
Stacking	86	82	80	87	80	83	84	87	84	86	83	91	92
Hard-voting	87	83	79	90	84	83	87	86	83	78	86	91	94
Soft-voting	89	82	79	89	84	83	87	84	83	78	86	90	94

The recall obtained by the models for all the feature selection techniques is described in Table 5. Mutual information was the best feature selection method. The recall obtained by the stack, hard-voting and soft-voting algorithms were 93%, 95% and 94%, respectively. The bat algorithm was the next best-performing model. The recall obtained by the stack, hard-voting and soft-voting models were 90%, 93% and 91%, respectively. The flower pollination algorithm performed well too. The recall obtained by the stack, hard-voting and soft-voting models were 86%, 90% and 90%, respectively. The recall obtained by the stack, hard-voting and soft-voting classifiers after using the Jaya algorithm was 87%, 91% and 90%, respectively. For further analysis, the best four feature selection techniques were considered. They are mutual information, bat algorithm, flower pollination algorithm and Jaya algorithm.

**Table 5:** Recall obtained by the classifiers for various feature selection methods (In %).

Algorithm	WO	SCA	PSO	JA	HHO	GWO	GA	FPO	FA	DE	CSA	BA	MI
Random forest	86	86	84	89	91	84	89	88	86	85	83	92	94
Logistic regression	84	78	82	86	87	82	85	85	87	84	84	86	88
Decision Tree	79	83	77	89	84	83	85	78	81	77	74	88	85
KNN	74	84	79	80	79	79	82	77	81	79	77	79	79
Adaboost	79	88	76	89	92	85	89	87	86	84	84	91	94
Catboost	87	89	80	91	94	85	90	88	86	88	83	91	96
Lightgbm	86	86	81	91	91	85	86	87	85	84	82	90	94
Xgboost	84	87	84	91	93	85	87	86	86	85	85	90	95
Stacking	84	83	81	87	81	85	86	86	87	85	82	90	93
Hard-voting	88	85	82	91	86	86	85	90	84	80	88	93	95
Soft-voting	87	83	81	90	86	86	85	90	84	80	87	91	94

The classification and the loss metrics are tabulated in Table 6. Mutual information performed the best among the four methods. The accuracy obtained for the stack, hard-voting and soft-voting classifiers were 90%, 95% and 94%, respectively. The bat algorithm was able to obtain excellent results too. The accuracies obtained by the stacking, hard-voting and soft-voting classifiers were 92%, 95% and 91%. The flower pollination algorithm performed relatively well. The accuracy obtained by the stacking, hard-voting and soft-voting classifiers were 87%, 85% and 86%. The accuracies obtained by the stack, hard-voting and soft-voting for the Jaya algorithm were 89%, 89% and 89%, respectively.

**Table 6:** Classification and loss metrics for the best four selection methods (In %).

Algorithm	Accuracy	F1-score	AUC	AP	MCC	Log loss	Jaccard score	Hamming loss
Mutual Information								
Random forest	91	90	0.97	0.98	0.80	3.19	0.86	0.09
Logistic regression	87	87	0.93	90.96	0.73	4.47	0.80	0.12
Decision Tree	87	87	0.9	0.9	0.73	4.34	0.81	0.12
KNN	78	77	0.77	0.79	0.53	7.54	0.69	0.21
Adaboost	89	89	0.96	0.98	0.77	3.70	0.83	0.10
Catboost	91	91	0.97	0.98	0.81	3.07	0.86	0.08
Lightgbm	90	89	0.97	0.98	0.78	3.45	0.85	0.1
Xgboost	91	91	0.97	0.98	0.81	3.07	0.86	0.08
Stacking	90	89	0.96	0.98	0.78	3.58	0.84	0.10
Hard-Voting	95	95	0.98	0.96	0.89	1.66	0.93	0.04
Soft-Voting	94	94	0.98	0.99	0.87	1.918	0.92	0.88
Bat Algorithm								
Random forest	90	89	0.95	0.97	0.77	3.45	0.86	0.1
Logistic regression	80	78	0.92	0.96	0.60	7.03	0.71	0.20
Decision Tree	87	85	0.92	0.95	0.71	4.47	0.82	0.12
KNN	77	76	0.79	0.85	0.53	7.00	0.695	0.22
Adaboost	88	87	0.95	0.97	0.73	4.09	0.83	0.11
Catboost	90	88	0.95	0.92	0.76	3.58	0.85	0.10
Lightgbm	92	91	0.95	0.97	0.81	2.81	0.88	0.08
Xgboost	92	91	0.95	0.98	0.81	2.68	0.89	0.07
Stacking	92	91	0.95	0.97	0.81	2.8	0.88	0.08
Hard-Voting	93	92	0.97	0.94	0.84	2.55	0.88	0.07
Soft-Voting	91	90	0.97	0.98	0.81	3.07	0.86	0.08
Flower Pollination Algorithm								
Random forest	87	86	0.95	0.97	0.73	4.47	0.81	0.12
Logistic regression	83	83	0.92	0.95	0.67	5.88	0.74	0.17
Decision Tree	83	82	0.9	0.94	0.65	6.01	0.74	0.17
KNN	77	76	0.77	0.8	0.51	6.05	0.67	0.23
Adaboost	84	84	0.93	0.96	0.67	5.32	0.77	0.15
Catboost	88	87	0.95	0.97	0.75	4.22	0.81	0.12
Lightgbm	89	88	0.95	0.97	0.76	3.83	0.83	0.11
Xgboost	86	85	0.94	0.96	0.69	4.98	0.78	0.144
Stacking	87	87	0.94	0.97	0.75	4.34	0.81	0.12
Hard-Voting	85	84	0.93	0.87	0.68	5.11	0.79	0.14
Soft-Voting	86	84	0.93	0.96	0.69	4.98	0.80	0.14
Jaya Algorithm								
Random forest	89	87	0.94	0.97	0.75	3.83	0.84	0.11
Logistic regression	80	78	0.92	0.96	0.60	7.05	0.71	0.20
Decision Tree	85	83	0.9	0.94	0.66	5.11	0.80	0.14
KNN	74	72	0.82	0.89	0.97	9.08	0.65	0.26
Adaboost	89	87	0.95	0.97	0.73	3.96	0.84	0.11
Catboost	89	87	0.94	0.97	0.74	3.96	0.84	0.11
Lightgbm	90	88	0.94	0.97	0.77	3.45	0.865	0.1
Xgboost	90	88	0.95	0.97	0.75	3.58	0.86	0.10
Stacking	89	88	0.94	0.97	0.75	3.70	0.855	0.10
Hard-Voting	89	88	0.95	0.89	0.76	3.96	0.82	0.1148
Soft-Voting	89	89	0.96	0.97	0.77	3.70	0.83	0.10

**Figure 7:**
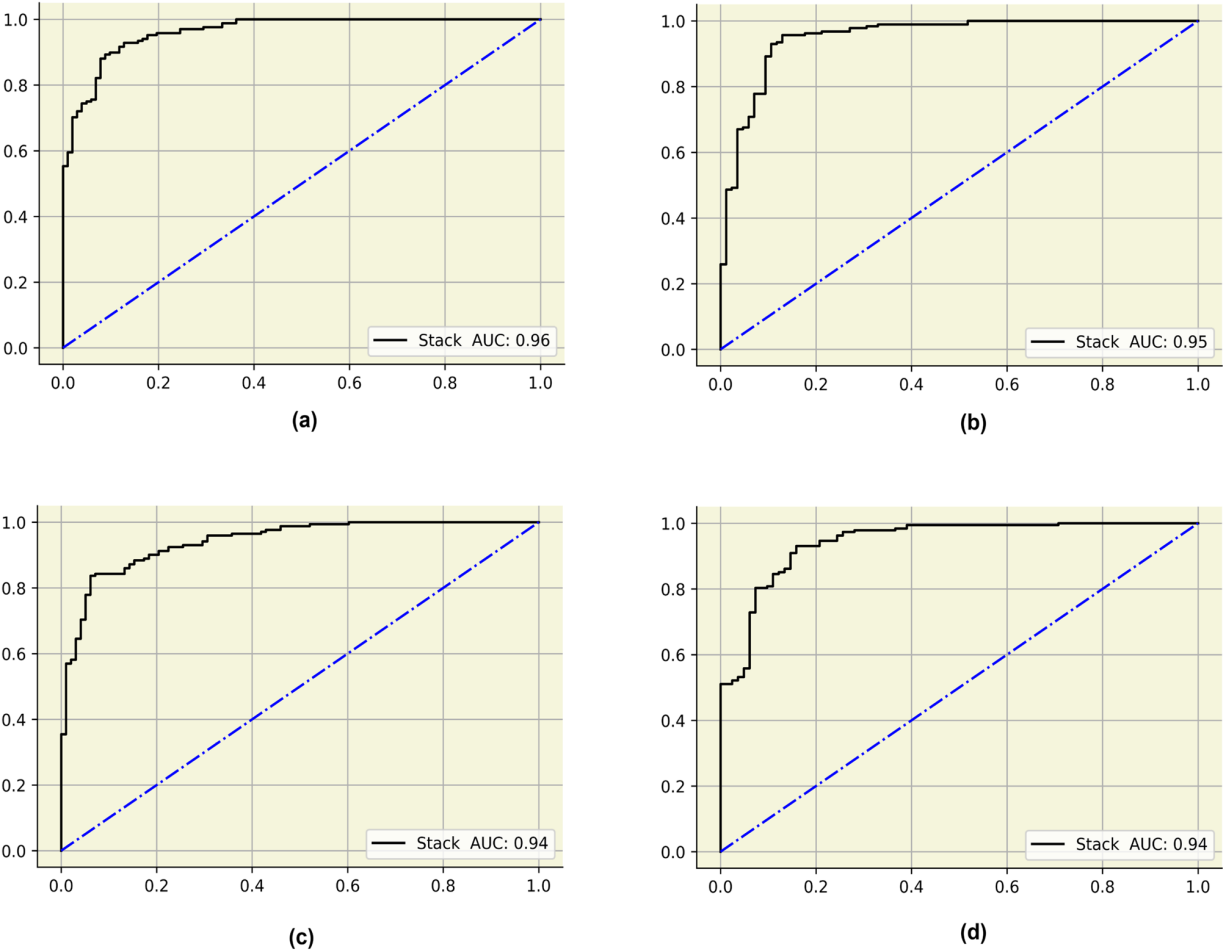
ROC curves for the stacked models. (**a**) MI (**b**) BA (**c**) FPA (**d**) JA.

**Figure 8:**
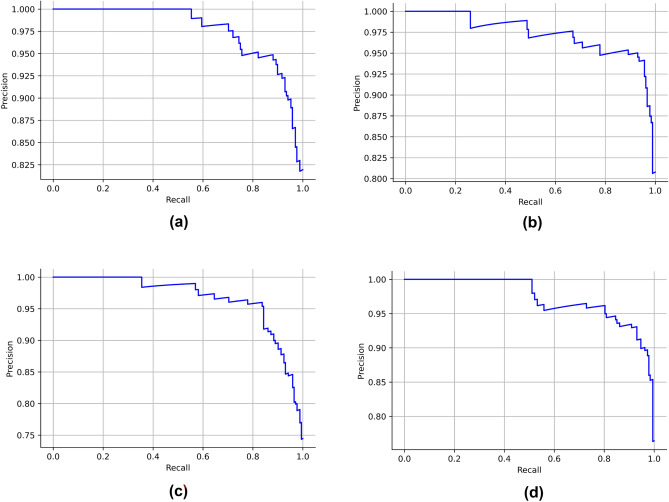
ROC curves for the stacked models. (**a**) MI (**b**) BA (**c**) FPA (**d**) JA.

The ROC curves for the stacked model for the four feature selection methods are depicted in Fig. 7. The AUC was maximum for the mutual information algorithm with 0.96. The precision-recall curves for the stacked classifiers for the four feature selection methods are described in Fig. 8. The stacked model obtained a maximum average precision of 0,98 after being trained on features chosen by mutual information.

**Figure 9:**
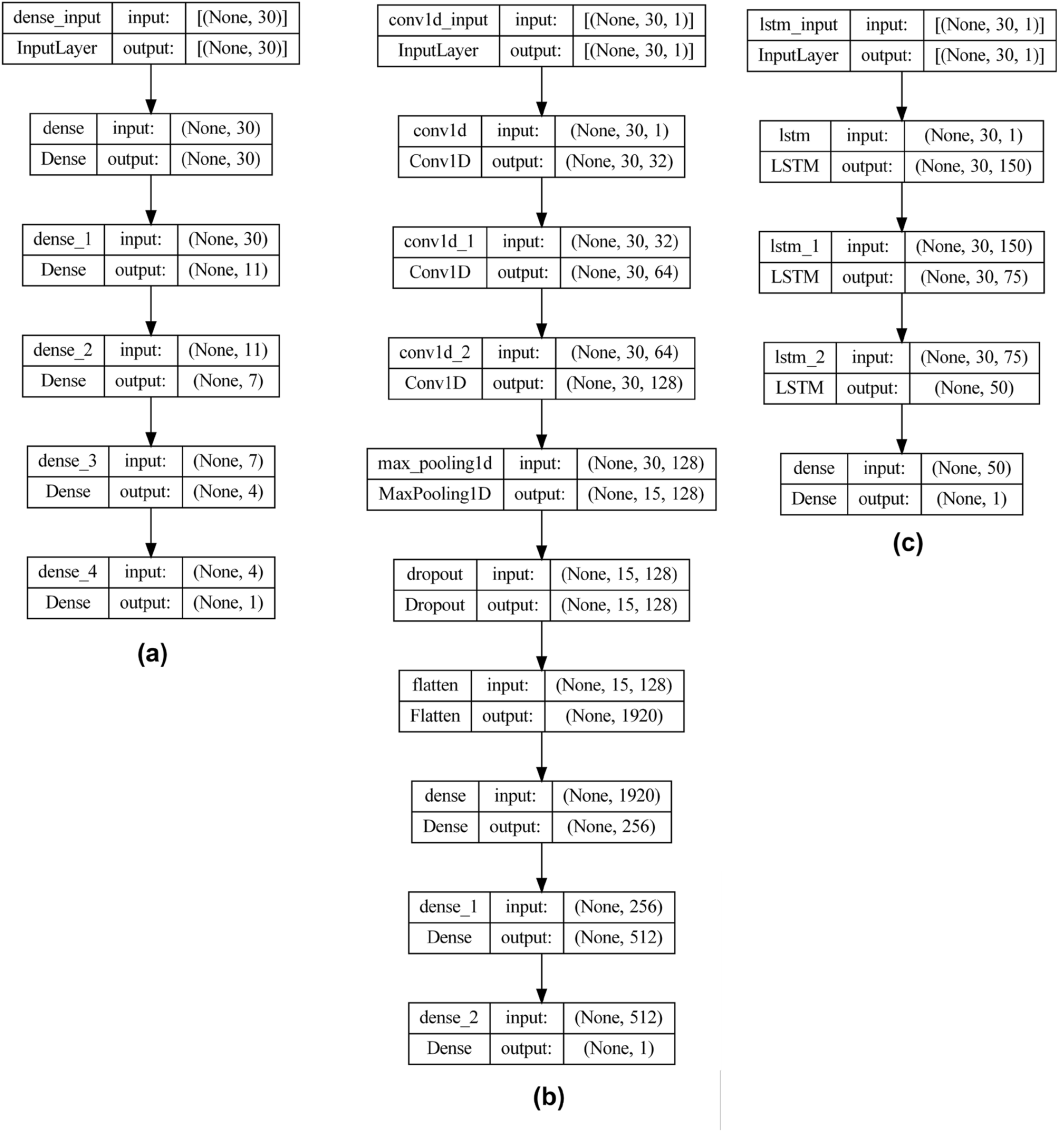
Architecture of the deep learning models. (**a**) DNN (**b**) 1D-CNN (**C**) LSTM.

Further, the results obtained by the machine learning models were compared with the deep learning models. DNN, 1D-CNN and LSTM were the classifiers used in this study. The model architecture of the deep neural networks is described in Fig. 9. For the DNN, five layers were considered. The number of neurons used was 30, 11, 7, 4 and 1. “Relu” was the activation function used for the input and hidden layers. The “sigmoid” activation function was used for the output layer. “Adam” was the optimizer, and “binary cross entropy” was the loss function used. A learning rate of 0.0001 was utilized, and the batch size was set to 10. The neural network was run for 750 epochs to establish reliable results.

For the 1D-CNN, we included layers such as conv1D, max pooling, drop out and flatten layers. The loss function was “binary cross entropy”, and “Adam” was the optimizer. The number of epochs and batch size were set to 10 each. A learning rate of 0.001 was utilized, and “leaky relu” was the activation function used for the input and hidden layers. “sigmoid” was the activation function used for the output layer.

The LSTM used four layers consisting of 150, 75, 50 and 1 neurons, respectively. The loss function used was “binary cross-entropy, and the optimizer was “Adam”. The batch size was set to 32.

**Figure 10:**
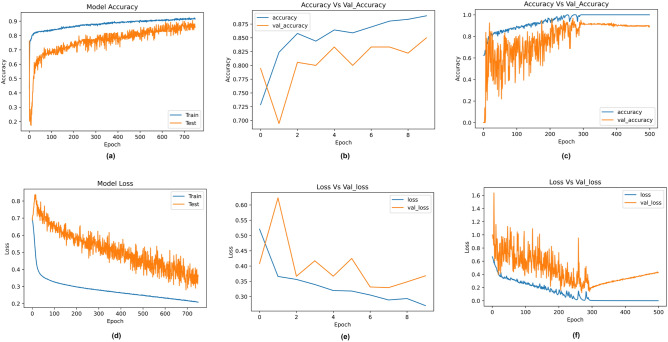
Accuracy and loss curves obtained by the deep learning classifiers. (**a**) Accuracy curve for DNN (**b**) Accuracy curve for 1D-CNN (**C**) Accuracy curve for LSTM (**d**) Loss curve for DNN (**e**) Loss curve for 1D-CNN (**f**) Loss curve for LSTM.

All three models were split into training and testing in the ratio of 80:20. The results obtained by the deep learning models are described in Table 7. Among the three, DNN performed the best, with an accuracy of 89%. 1D-CNN and LSTM obtained accuracies of 85% and 83%, respectively. The accuracy and loss curves for the models are depicted in Fig. 10. From the figure, the results obtained by the models are reliable and not overfitting.

**Table 7:** Classification and loss metrics obtained by the deep learning models.

Algorithm	Accuracy	Precision	Recall	F1-score	AUC	Hamming loss	Jaccard score	MCC	Log loss
DNN	89	88	87	88	97	0.11	0.83	0.75	3.96
1D-CNN	85	83	84	83	94	0.15	0.79	0.66	5.18
LSTM	83	79	81	80	88	0.17	0.77	0.60	5.88

### Explainable artificial intelligence

In this study, five XAI methods: SHAP, LIME, QLattice, Eli5 and Anchor have been used to make the models more interpretable. We chose the stacked model for interpretation since they obtained good results and are generally reliable. Deep learning classifiers were not considered since many explainers do not support deep learning algorithms today. Further, machine learning algorithms performed better than deep learning models in this study. This is normal in artificial intelligence applications since deep learning models perform better only with comprehensive data.

**Figure 11:**
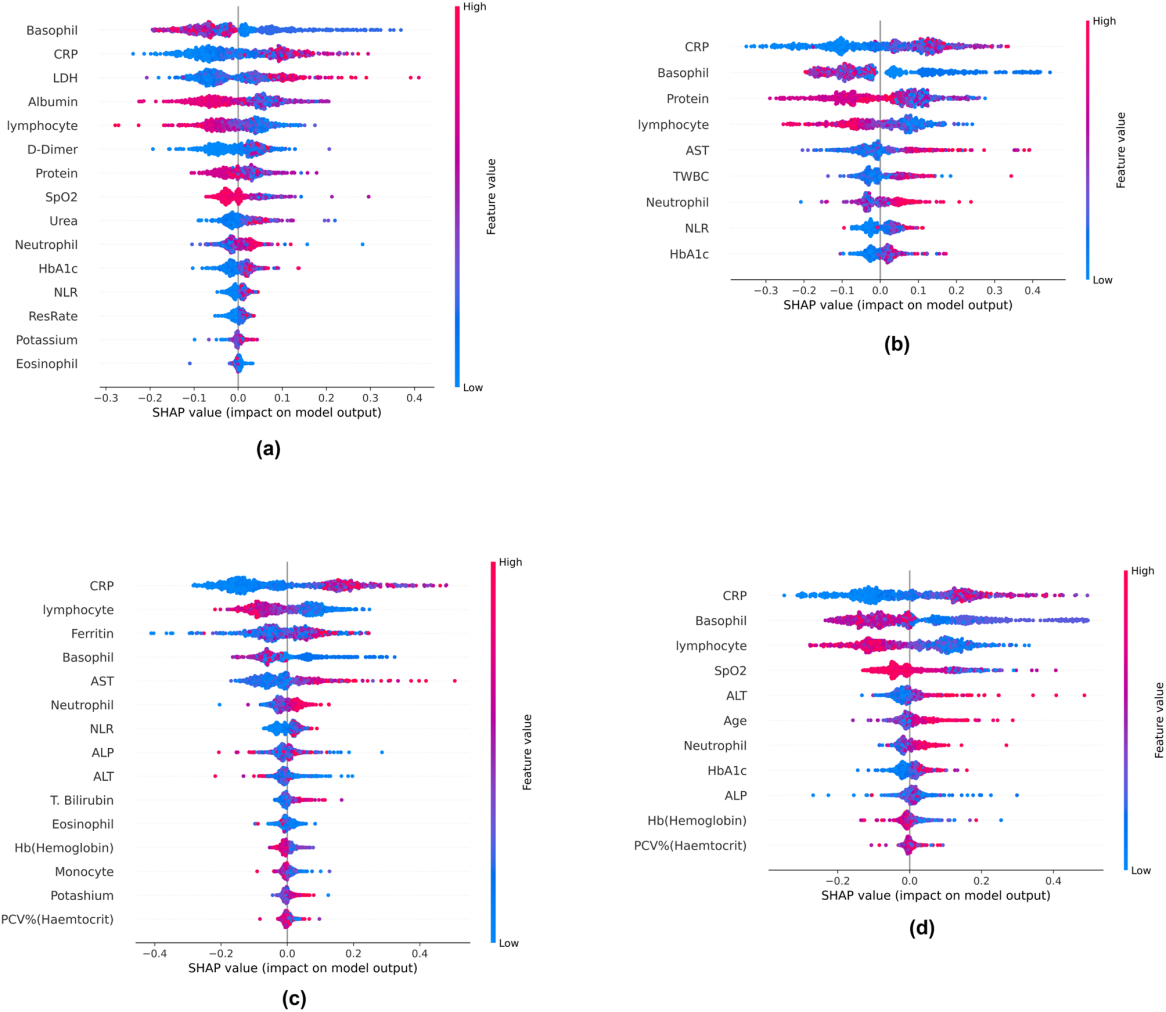
Global SHAP interpretation using beeswarm plots. (**a**) MI (**b**) BA (**c**) FPO (**d**) JA.

SHAP is a widely used XAI technique that makes global and local interpretations^42^. SHAP uses game and probability theory to understand the impact of each attribute. The global interpretation of the models is explained using beeswarm plots as described in Fig. 11. A hyperplane separates the non-severe (left) and severe classes (right). Red indicates a higher value, and blue indicates a lower value. The markers are also arranged based on their importance (The best feature remains at the top). The figure shows that the most important markers are basophils, CRP, LDH, lymphocytes, albumin, protein and ferritin. CRP, LDH and Ferritin levels increased in severe COVID-19 patients. Basophils, lymphocytes, albumin and protein levels decreased in severe COVID-19 patients.

**Figure 12:**
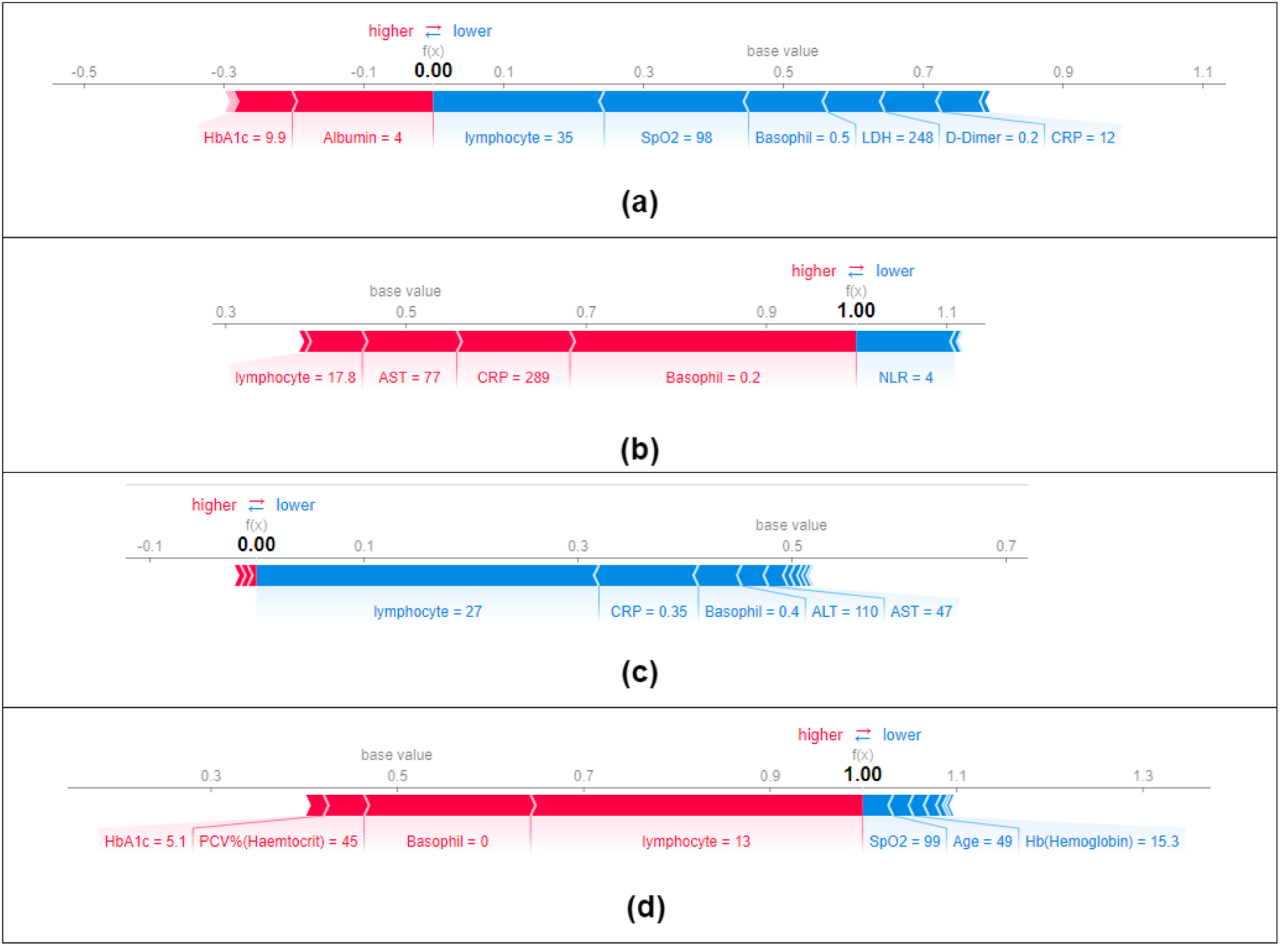
Local SHAP interpretation using force plots. (**a**) MI (**b**) BA (**c**) FPO (**d**) JA.

Local interpretations can be explained using the SHAP force plot, as shown in Fig. 12. Figure 12a,c indicate a non-severe prognosis. It can be seen that markers such as lymphocytes, SPO2, basophils and CRP are pushing the predictions towards a non-severe prognosis. Figure 12b,d indicate a severe COVID-19 prognosis. Markers such as CRP, AST, basophils and lymphocytes push the predictions towards severe COVID-19.

**Figure 13:**
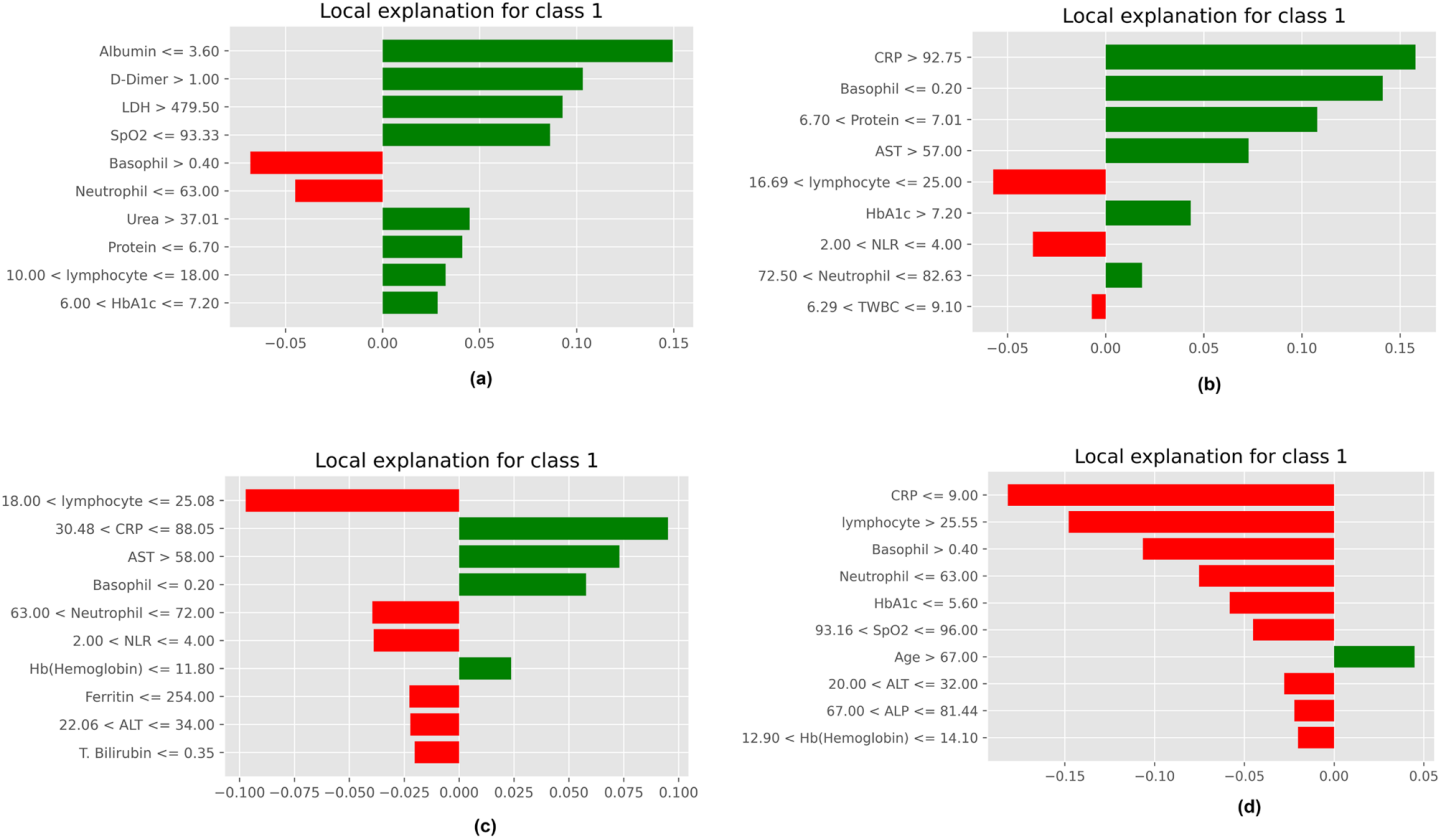
Model explainablity using LIME. (**a**) MI (**b**) BA (**c**) FPO (**d**) JA.

LIME is another explainer used to make local interpretations^43^. It uses a model-agnostic approach (It works for most ML models). It uses a ridge regression model and kernels such as Gaussian and RBF to explain the predictions. The LIME interpretations are depicted pictorially in Fig. 13. Figure 13a,b predict a severe prognosis, and Fig. 13c,d indicate a non-severe prognosis. The attributes are also arranged based on the descending order of their importance. The figure shows that the most important markers are albumin, D-Dimer, LDH, CRP, basophils, protein, AST, SPO2 and lymphocytes.

**Figure 14:**
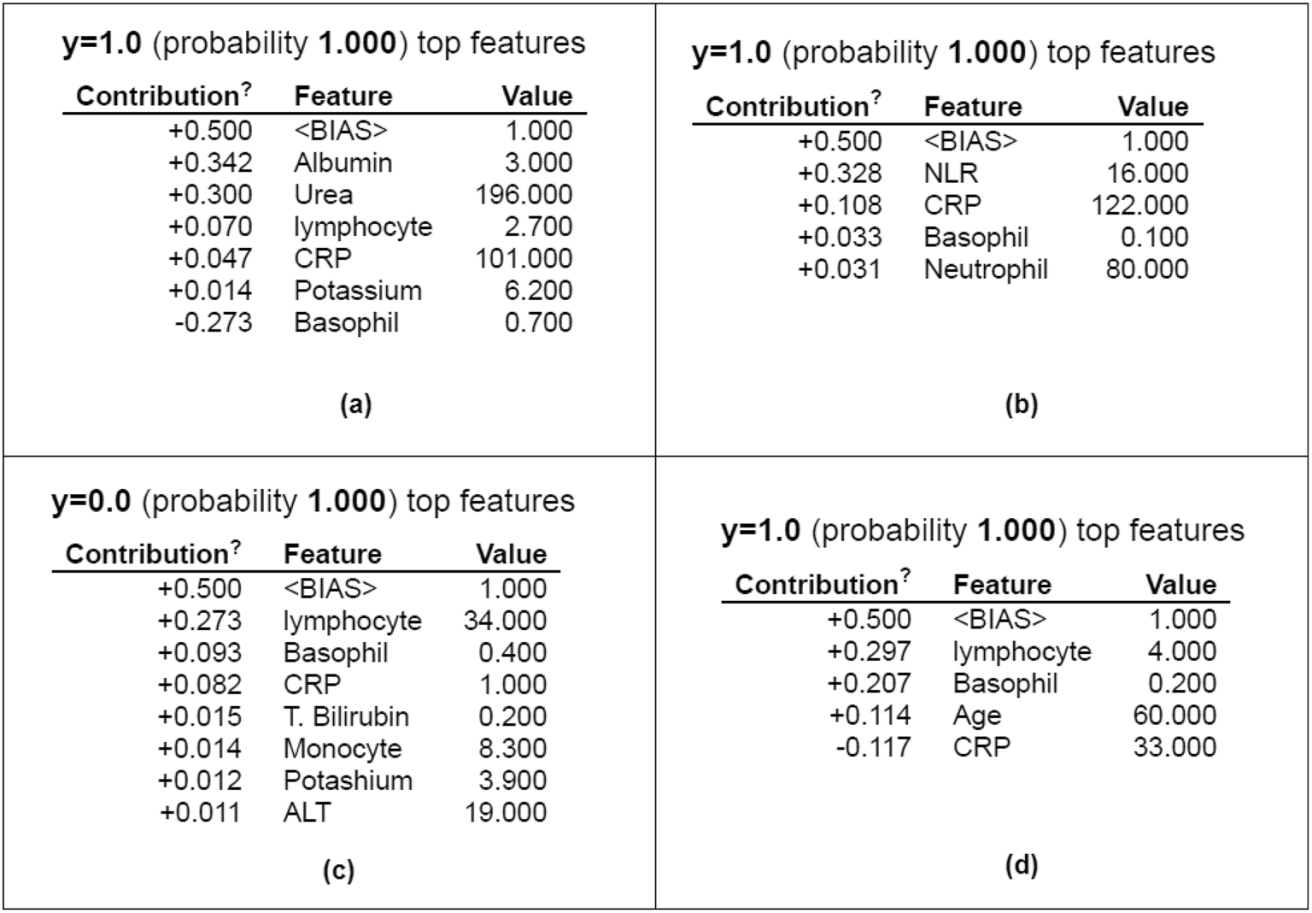
Model explainablity using Eli5. (**a**) MI (**b**) BA (**c**) FPO (**d**) JA.

Eli5 is yet another method to demystify predictions^44^. It is a python package and is highly used with tree-based classifiers. Figure 14 depicts Eli5 predictions, and according to it, the most essential attributes are albumin, urea, lymphocytes, CRP, NLR, and basophils count. This explainer considers the “bias” (error rate).

**Figure 15:**
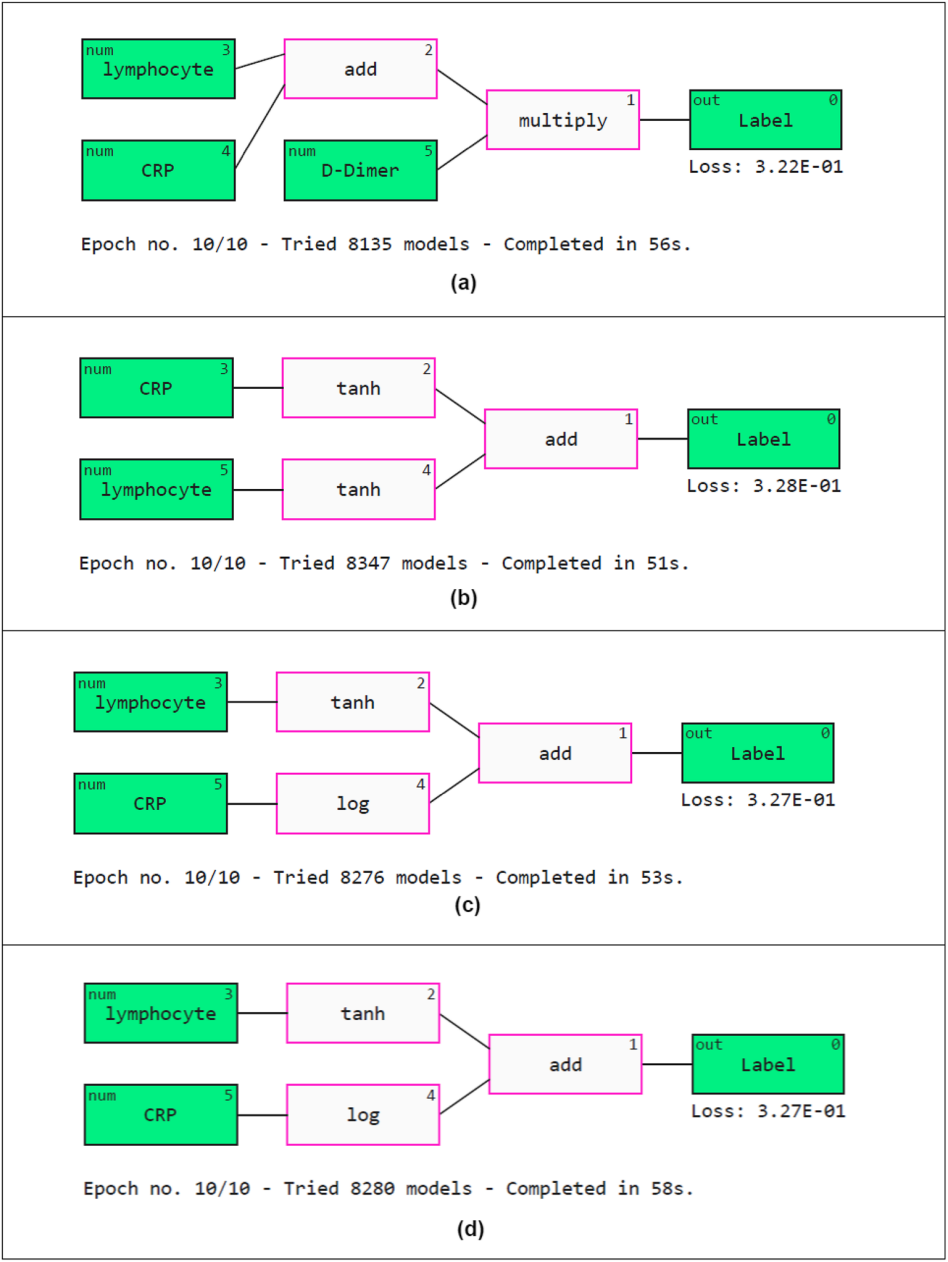
Model explainablity using QLattice. (**a**) MI (**b**) BA (**c**) FPO (**d**) JA.

Abzu developed the QLattice explainer^45^. It uses quantum computing and symbolic regression to explain the predictions. QLattice trains the models to understand the variation in data. The input attributes are called registers. A collection of registers is termed a QGraph. Every QGraph has a set of nodes (registers) and activation functions. Activation functions such as add, multiply, log, sine, tanh and Gaussian are generally used. The QGraphs are described in Fig. 15. It can be seen that the most important markers are lymphocytes, CRP and D-Dimer.

Anchor is an XAI technique that uses rules and conditions^46^. The strength of an anchor is measured using its precision and coverage. Precision defines the accuracy of the anchor. Coverage determines how many instances utilize the same conditions. The anchors for non-severe and severe cases are described in Table 8. The most important markers are basophils, albumin, lymphocytes, CRP, D-Dimer, neutrophils, protein and NLR.

**Table 8:** Model explainablity using Anchor.

Prediction	Anchor	Precision	Coverage
Mutual Information			
Non-severe COVID-19	Basophil > 0.20 and Albumin > 4.00	0.9	0.33
Non-severe COVID-19	Basophil > 0.22 and Lymphocyte > 26	0.94	0.18
Severe COVID-19	CRP > 79.41 and Neutrophil > 81.58	0.99	0.15
Severe COVID-19	D-Dimer < = 1.00 and Albumin > 4.00	0.86	0.46
Bat Algorithm			
Non-severe COVID-19	CRP < = 10.25 and Lymphocyte > 25.00	0.93	0.12
Non-severe COVID-19	Basophil > 0.40 and Lymphocyte > 25.00	0.94	0.06
Severe COVID-19	CRP > 92.75 and Neutrophil > 82.63	0.93	0.13
Severe COVID-19	Basophil < = 0.20 and Protein < = 7.01	0.85	0.30
Flower Pollination Algorithm			
Non-severe COVID-19	CRP < = 30.48 and NLR < = 2.0	0.85	0.24
Non-severe COVID-19	CRP < = 10.49 and Lymphocyte > 25.08	0.90	0.12
Severe COVID-19	CRP > 68.05 and NLR > 8.14	1	0.13
Severe COVID-19	CRP > 30.40 and Lymphocyte < = 18.00	0.91	0.33
Jaya Algorithm			
Non-severe COVID-19	CRP < = 9.00 and Neutrophil < = 63.00	0.89	0.12
Non-severe COVID-19	Basophil > 0.20 and Lymphocyte > 25.55	0.95	0.20
Severe COVID-19	Basophil < = 0.20 and SpO2 < = 93.16	0.97	0.17
Severe COVID-19	CRP > 90.25 and Lymphocyte < = 9.60	1	0.14

Five XAI techniques have been utilized and their findings are similar. The most important markers that can predict a patient's severity are CRP, lymphocytes, basophils, albumin, D-Dimer, NLR, and neutrophils.

## Discussion

This research used multiple machine learning algorithms to predict severe COVID-19 cases in advance so that appropriate treatments could be provided for vulnerable patients. To demystify the predictions, five heterogenous XAI techniques were used. Doctors and medical professionals can easily understand the variation in the markers provided by the explainers. This decision support system can be setup in various medical facilities to aid healthcare workers. In developing countries, this application can be used to make judicious use of essential medical assets such as ICU beds, ventilators and medicines. The models can also be utilized to present a second opinion to the doctors.

Fourteen feature selection methods were utilized and we chose the best four for further analysis. They are mutual information, bat algorithm, flower pollination algorithm and Jaya algorithm. A maximum accuracy of 95% was obtained by the mutual information algorithm. The F1-score, AUC and AP were 94%, 0.98 and 0.99. When the bat algorithm was utilized, a 93% accuracy was obtained. The F1-score, AUC and AP were 92%, 0.97 and 0.94. When the flower pollination algorithm was used, an accuracy of 89% was obtained. The F1-score, AUC and AP were 88%, 0.95 and 0.97. When the Jaya algorithm was utilized, a 90% accuracy was obtained. The F1-score, AUC and AP were 88%, 0.95 and 0.97. Most machine learning models performed relatively well.

Several markers showed variation between the two cohorts. Among all, CRP was chosen by all the XAI techniques. CRP levels increased in severe COVID-19 patients in this study^47^. Lymphocyte levels decreased in severe COVID-19 patients. Lymphopenia has been commonly recorded in patients when their conditions deteriorate^48^. This research also observed Basopenia in severe COVID-19 patients^49^. Low serum albumin has been associated with severe COVID-19^50^. This marker variation was also observed in this study. D-Dimer has already been an important marker in predicting COVID-19^51^. Elevated D-Dimer levels were found in the severe COVID-19 cohort. NLR is a vital marker which has already been utilized to diagnose and predict severity in patients. NLR levels elevates in severe COVID-19 patients^52^. The same trend has been observed in this research. Spo2 levels decreased rapidly in the severe COVID-19 cohort. Lower oxygen levels can seriously threaten COVID-19 patients since it causes hypoxia^53^. Age was also observed to be an important factor in predicting COVID-19 severity. Older patients were more vulnerable to experiencing severe symptoms^54^. The above markers can be monitored carefully to prevent a fatal prognosis.

Various machine researches have been conducted to predict the severity of COVID-19. Raman et al.^55^ used machine learning to predict COVID-19 severity during hospital admission. Patient data was collected from the University of Texas. The random forest obtained a sensitivity of 72% and a specificity of 78%. Their model could predict the severity within six hours of hospital admission. Ershadi et al.^56^ used image and clinical data to predict COVID-19 severity. A fuzzy-based classifier was developed to forecast severe cases. Two datasets were used, and the accuracies obtained for them were 92% and 90%, respectively. Chest X-ray images and clinical data were used to predict COVID-19 severity in another research^57^. 930 COVID-19 patients from Italy were considered for this research, and the stacking classifier achieved an accuracy of 89.03%. The most important markers were LDH, CRP, age, WBC and SpO2. Bello et al.^58^ used clinical markers and omics to predict COVID-19 severity. The model obtained an accuracy of 91.6%. The most important markers were LDH, albumin, creatinine, lymphocytes, neutrophils and potassium.

However, no articles that use five different XAI techniques to predict COVID-19 severity exist. Explainers such as Anchor, QLattice and Eli5 have been rarely used in medical machine learning. There are some limitations in our study too. The data collected was from a single geographical territory (India). Multiple datasets from different sources must be considered to make the classifiers more reliable. This research made exclusive use of supervised learning. Unsupervised learning and reinforcement learning algorithms were not considered. Graphical processing units (GPU) increase computational speed while training. However, they were not used in this study. We had divided the dataset into training and testing and had performed cross validation. However, we could not test the data on real-time patients as our study was retrospective. Prospective study can be conducted in the future to test real patients’ prognosis. Our machine learning models could also be used for other diseases and public health issues^59–65^.

## Conclusions

XAI is a part of machine learning, generally used to demystify the predictions made by the classifiers. In this study, we used several supervised learning algorithms and XAI techniques to predict the COVID-19 severity in advance. The patients vulnerable to severe COVID-19 symptoms can be identified early, and appropriate treatments can be provided to save them. Various patterns and trends in the clinical markers were observed using descriptive statistics in the initial part of this research. Multiple feature selection techniques, including nature-inspired algorithms, were utilized to select the most crucial parameters. Several algorithms, such as bagging, boosting, stacking, voting and state-of-art deep learning, were used to make accurate predictions. The mutual information algorithm proved to be the most efficient feature selection technique obtaining a maximum accuracy of 95%. Five heterogeneous XAI algorithms such as, SHAP, LIME, QLattice, Eli5 and Anchor, have been used to understand the classification predictions. According to them, the most essential marker was CRP. Other markers such as D-Dimer, lymphocytes, neutrophils, albumin and basophils were also crucial. The classifiers can be utilized as a decision support system in various hospitals for prediction. The models can be used to predict the COVID-19 severity in advance. It can also aid the medical professionals and can offer them a second opinion. The algorithms can also be used for a rapid diagnosis too.

In the future, cloud-based models can be deployed. They can easily store both the data and code more efficiently. High-end GPUs can be utilized to train deep learning algorithms. Other diagnostic methods, such as rapid antigen tests, chest X-rays and genome sequencing, can be combined suitably. Prognosis can be predicted for various COVID-19 variants. Electronic health records from multiple hospitals across various countries can be combined before training the models. Other deep learning techniques such as fuzzy ensembling techniques could be utilized.

## Data Availability

Data will be made available by Mr. Krishnaraj Chadaga after obtaining prior permission from Manipal Academy of Higher Education.
